# Sociodemographic and Health-Related Predictors of Anxiety and Fear of COVID-19 in the Prison Population: Cross-Sectional Study

**DOI:** 10.2196/88699

**Published:** 2026-11-09

**Authors:** Francisco Javier González-Riera, Regina Allande-Cussó, Luis El Khoury-Moreno, Murat Yıldırım, Krzysztof Goniewicz, Azahara Ruger-Navarrete, Beatriz Mérida-Yáñez, Julio Torrejón-Martínez, Javier Fagundo-Rivera, Juan Gomez-Salgado

**Affiliations:** 1 Surveillance, Health Promotion and Prevention Unit, Jaén and Jaén Sur Health District, Andalusian Health Service (SAS), Jaén, Spain; 2 Department of Nursing, Faculty of Nursing, Physiotherapy and Podiatry, Universidad de Sevilla, Calle Avenzoar 6, Sevilla, Andalusia, 41009, Spain, 1 954557906; 3 Department of Stomatology, Faculty of Odontology, Universidad de Sevilla, Sevilla, Andalusia, Spain; 4 Department of Psychology, Faculty of Science and Letters, Ağrı İbrahim Çeçen University, Ağrı, Türkiye; 5 Psychology Research Center, Khazar University, Baku, Azerbaijan; 6 Department of Psychology, Faculty of Arts and Sciences, Haliç University, İstanbul, Türkiye; 7 Department of Security Studies, Polish Air Force University, Dęblin, Lublin, Poland; 8 Department of Nursing, Faculty of Health Sciences of Ceuta, Universidad de Granada, Ceuta, Spain; 9 Department of Medicine and Public Health, Faculty of Labour Sciences, Universidad de Huelva, Huelva, Andalusia, Spain; 10 Safety and Health Postgraduate Program, Universidad de Especialidades Espíritu Santo, Guayaquil, Ecuador

**Keywords:** prisoners, prisons, COVID-19, anxiety, fear, psychological distress, pandemics, mental health

## Abstract

**Background:**

The COVID-19 pandemic generated a notable emotional impact on the general population, yet its effects on incarcerated individuals have been far less explored, despite the particular vulnerabilities associated with prison environments.

**Objective:**

This study aimed to assess levels of anxiety and fear related to COVID-19 among incarcerated adults in Spain and identify sociodemographic and health-related factors associated with greater emotional distress.

**Methods:**

A cross-sectional study was conducted between May 2022 and February 2023 among 711 inmates from 3 Spanish prisons using a nonprobability convenience sampling strategy. Anxiety and fear related to COVID-19 were assessed using the Anxiety and Fear of COVID-19 Assessment Scale (AMICO)–Inmates scale, an adaptation of the original AMICO, developed for the general Spanish population, and subsequently validated for use among prison inmates. Descriptive analyses, bivariate comparisons, and categorical regression (CATREG) were performed to examine factors associated with the total AMICO score.

**Results:**

The mean AMICO score was 5.58 (SD 2.55), indicating high levels of anxiety and fear related to COVID-19. In the bivariate analyses, significant differences were observed according to sex, having children, sharing a cell, and the presence of chronic illness. The CATREG model explained 17.4% of the variance in AMICO scores (*R*^2^=0.174; *P*<.001). Female sex (β=0.214; *P*<.001), sharing a cell (β=0.112; *P*=.001), and chronic illness (β=0.086; *P*=.01) were independently associated with higher levels of anxiety and fear.

**Conclusions:**

Incarcerated individuals experienced high levels of anxiety and fear related to COVID-19. Women, inmates sharing a cell, and those with chronic illnesses appeared particularly vulnerable to emotional distress. These findings highlight the importance of incorporating targeted psychological support and mental health strategies into prison preparedness plans for future public health emergencies.

## Introduction

The psychological impact of infectious disease outbreaks on the general population has been extensively documented over the past decades. Studies following epidemics such as severe acute respiratory syndrome, influenza A and virus subtype H1N1, and Ebola have consistently reported increased mental health morbidity, particularly anxiety and depression, especially during the early stages of these events [1-3]. When the COVID-19 pandemic emerged, researchers rapidly turned their attention to its psychological effects on emotional well-being and sense of coherence [4-6], identifying high levels of anxiety from the outset and emphasizing the urgent need for targeted mental health interventions. Alongside this, protective factors such as access to adequate health information and the adoption of preventive behaviors were highlighted [7], while evidence further pointed to the central role of information exposure in shaping both anxiety and depression and promoting adaptive protective responses [3,8,9].

Central to understanding these psychological dynamics is the distinction between two closely related but conceptually distinct constructs. Fear is conceptualized as an emotional response to a real or perceived imminent threat, typically associated with immediate defensive reactions and autonomic activation, whereas anxiety refers to a more future-oriented state characterized by anticipation of potential threat, often accompanied by excessive worry and heightened physiological arousal in the absence of immediate danger [10]. In the context of COVID-19, the literature has described a close association among high infection rates, fear, and pandemic-related morbidity and mortality, with elevated fear levels shown to trigger maladaptive behaviors and emotional responses [11].

These effects, while pervasive across the general population, take on particular significance in closed institutional settings such as prisons, where structural conditions and restricted autonomy create heightened psychological vulnerability [12]. The Spanish prison system classifies inmates into three custody grades that fundamentally determine their living conditions. The first grade, or closed regime, involves individual cell confinement with minimal social interaction. The second grade (the most prevalent) corresponds to an ordinary regime in which inmates typically share cells and have daily access to common areas and structured activities. The third grade, a semiopen regime, allows nearly normalized contact with the outside world [13]. The Spanish prison system comprises 65 prisons, 2 psychiatric hospitals, and 13 open facilities, forming a modern and functionally integrated network designed to implement custodial sentences, security measures, and pretrial detention within largely self-contained institutional settings [14]. These differences in confinement levels and social support are critical to understanding how pandemic-related restrictions may have differentially affected anxiety and fear across the incarcerated population [15,16].

During the COVID-19 pandemic, Spanish prisons implemented strict containment measures, including the complete suspension of family visits, prolonged cell confinement amounting to extended solitary isolation, and the cancellation of all group activities such as workshops and outdoor exercise [17]. While these measures were designed to curb transmission, they simultaneously led to the abrupt elimination of social support and daily routine, two cornerstones of emotional stability in custodial settings. It is plausible that such conditions intensified feelings of loneliness, uncertainty, and hopelessness, thereby elevating levels of anxiety and fear in this population. Notably, these strategies differed markedly from those adopted in other countries, where compensatory measures such as video calls or controlled social interactions were frequently maintained [18-21], highlighting the need to contextualize observed psychological outcomes within the specific framework of the Spanish penitentiary system.

The psychological consequences of these conditions have been well documented in the broader prison literature. Social interaction is a fundamental determinant of psychological well-being, and prolonged social isolation has been consistently associated with adverse mental health outcomes, including anxiety, depression, emotional dysregulation, cognitive disturbances, self-harm, and suicide risk [22-28]. These effects may be particularly pronounced in prison settings, where restrictions imposed during the COVID-19 pandemic further limited interpersonal contact and access to social support [19-21]. During this period, fear of infection, the impact of social distancing, the interruption of family visits, and the reduction of mental health services appear to have compounded these preexisting vulnerabilities [19]. Furthermore, poor communication by prison staff regarding pandemic measures has been identified as an additional source of stress and anxiety among inmates [20], while reports indicate that prisoners placed in isolation typically spent between 22.5 and 24 hours per day in their cells, sometimes for months or even years without a defined end point [21]. The high prevalence of preexisting mental health conditions in this population, including mood disorders, substance use disorders, anxiety, depression, and psychosis [29], as well as a family history of mental health problems [30], further renders incarcerated individuals particularly susceptible to the psychological sequelae of pandemic-related restrictions. More broadly, prison environments may amplify the psychological consequences of public health emergencies owing to overcrowding, limited autonomy, reduced access to health care resources, and the concentration of individuals with preexisting vulnerabilities [12,31-33]. Recent research conducted after the acute phase of the pandemic has continued to document substantial psychological distress among incarcerated populations, with persistent symptoms of anxiety, depression, stress, and fear linked to infection risk, restricted social contact, and institutional uncertainty [34,35]. Within this context, fear and anxiety emerged as common psychological responses to perceived threats associated with infection, uncertainty, and concerns about personal and family well-being [36-38].

To assess these psychological responses, validated instruments have been developed and adapted for use in diverse populations. The Fear of COVID-19 Scale has been validated in English [39], Italian [40], and Spanish [11]. Building on this work, Gómez-Salgado et al [41] designed the Anxiety and Fear of COVID-19 Assessment Scale (AMICO, for its acronym in Spanish), beginning with the 10 initial items evaluated by a panel of experts in the study by Ahorsu et al [39] and incorporating 8 additional items to capture COVID-19–specific anxiety alongside the fear construct. The resulting 16-item instrument demonstrated reliability as a screening tool [41] and was subsequently validated in a prison population, where it showed a consistent factor structure and high internal reliability, supporting its use in custodial contexts [42].

Despite this growing body of evidence, studies specifically examining anxiety and fear related to COVID-19 among incarcerated populations remain scarce, particularly in Spain. Given the heightened vulnerability of prison settings during public health emergencies, further research is warranted to better identify these emotional responses in this underserved population. Therefore, the objective of this study was to evaluate the levels of anxiety and fear related to COVID-19 in an adult prison population in Spain following the validation of the AMICO–Inmates through a field study conducted among incarcerated individuals.

## Methods

### Design

A descriptive cross-sectional study was conducted using questionnaire-based data collection. The reporting of this study adhered to the STROBE (Strengthening the Reporting of Observational Studies in Epidemiology) (Checklist 1) guidelines for cross-sectional studies [43].

### Participants

The study population consisted of inmates housed in Spanish penitentiary institutions. The total prison population in Spain has been estimated at 49,998 individuals [44]. The sample size was determined using 2 complementary criteria. First, considering the target population, a minimum sample of 450 participants was calculated using a 95% confidence level, 3% precision, and an anticipated attrition rate of 25%. Second, given that the study involved administration of the 16-item AMICO–Inmates, the recommendation of at least 10 participants per item was also considered, resulting in a minimum required sample of approximately 200 participants for stable psychometric and multivariable analyses [45]. The more conservative population-based criterion (n=450) was adopted as the minimum target sample size.

The Spanish prison system comprises 65 prisons. Given the national scope and logistical constraints associated with data collection in correctional environments, a nonprobability convenience sampling strategy was used. Specifically, the selection of the 3 participating institutions (Albolote, Granada; Jaén; and Córdoba) was based on institutional accessibility, administrative authorization, and willingness to participate, which are common constraints in prison-based research.

Inclusion criteria were being aged 18 years or older, being incarcerated in one of the participating institutions during the data collection period, being able to understand and complete the questionnaire, and providing informed consent to participate. No additional exclusion criteria were established other than refusal to participate or inability to provide informed consent. All inmates present in the participating modules on the days of data collection who met the inclusion criteria were invited to participate voluntarily.

A total sample of 711 participants was ultimately obtained, exceeding the minimum sample size required by both calculation approaches. The calculated sample size represented the minimum number of participants required to ensure adequate statistical power. As recruitment continued throughout the study period, all eligible individuals who met the inclusion criteria and agreed to participate were included, resulting in the final sample being larger than the minimum required.

### Variables

The questionnaire collected the following sociodemographic and health-related variables: sex, age, province of residence, marital status, number of children, income, degree of sentence, number of people sharing the cell, perceived health status, presence of chronic disease, and substance use. Scale-based variables were also included, among them the AMICO–Inmates score. Due to feasibility constraints associated with data collection in correctional settings and the need to minimize respondent burden, information regarding previous mental health diagnoses and COVID-19 vaccination status was not collected.

The AMICO–Inmates is a validated instrument designed to assess anxiety and fear specifically related to the COVID-19 pandemic in incarcerated populations [42]. It comprises 16 items grouped into 2 correlated dimensions (anxiety and fear) using a 10-point Likert-type response format ranging from 1 (“strongly disagree”) to 10 (“strongly agree”). Validation in the Spanish prison population demonstrated a robust bifactor structure, adequate unidimensionality (percentage of uncontaminated correlations=0.52; explained common variance=0.64; omega hierarchical=0.82), and excellent reliability (Cronbach α=0.95; McDonald ω=0.951), supporting its internal consistency and sensitivity as a screening tool in this context [42]. Although the scale comprises 2 correlated dimensions, previous validation studies have supported the calculation and interpretation of a global score. Therefore, the total AMICO score, obtained as the mean of all item responses, was used as the dependent variable in the present analyses [42].

According to the validation study, interpretation of AMICO–Inmates scores is based on percentile-derived categories established from the distribution of the validation sample: low (0.0‐3.4 points), moderate (3.5‐5.5 points), and high (>5.5 points) levels of anxiety and fear related to COVID-19. These categories showed statistically significant differences during the validation process (all Mann-Whitney *U* tests; *P<*.001) and were proposed as interpretative thresholds for the instrument [42].

### Procedure

The necessary permits to conduct the research were obtained from the General Secretariat of Penitentiary Institutions of the Ministry of the Interior, as well as from the management teams of each penitentiary institution.

Access to the centers was facilitated by the medical and/or treatment subdirectorate of each facility, which coordinated communication between the research team and the health care staff, educators, and correctional officers responsible for the modules where data collection took place. The study used a nonprobability convenience sampling approach. Participants were recruited through official and authorized channels, with prison staff assisting in the organization of data collection sessions and the distribution of questionnaires.

Staff were previously instructed by the research team regarding the study aims and procedures. In each module, inmates were gathered in common areas, where members of the research team, assisted by prison staff, administered the paper-based questionnaires and provided standardized instructions while resolving any doubts.

Although the questionnaire was hetero administered by trained prison staff under researcher supervision, strict confidentiality was ensured at all times. Inmates were informed that participation was voluntary, responses were anonymous, and completed questionnaires would be handled exclusively by the research team with no access by prison staff or institutional authorities, thereby minimizing social desirability bias and ensuring privacy in the provision of answers.

Sampling began at the Jaén prison on May 26 and 27 and June 1, 2022. It continued at the Albolote prison (Granada) on September 23, 26, and 29 and October 3 and 5, 2022. The final sampling day was February 23, 2023, in Córdoba.

### Data Analysis

The SPSS Statistics software (version 26; IBM Corp) was used for univariate and bivariate descriptive analysis. Normality of the score distribution was assessed using the Kolmogorov-Smirnov test, which yielded a *P* value of .01, indicating a nonnormal distribution. Accordingly, nonparametric contrast tests were performed, including the Mann-Whitney *U* test and the Kruskal-Wallis test. The Kendall tau-b coefficient was used to examine the correlation between quantitative variables.

To examine the relationship between anxiety and fear levels (total AMICO score) and the remaining variables, a categorical regression analysis (CATREG) was performed given that these variables were qualitative in nature. The CATREG analysis includes key features of classic regression analysis, such as the coefficient of determination (*R*^2^), the ANOVA in regression, and the significance of the parameters. For the analysis, the optimal scaling option in SPSS was selected. Categorical regression (CATREG) results are reported using standardized coefficients (β), *F* statistics, and associated *P* values in accordance with the standard output generated by the CATREG procedure in SPSS. Variables were selected for inclusion in the CATREG model solely based on statistical significance in the bivariate analyses (*P<*.05). Variables meeting this criterion were subsequently entered into the model to identify independent predictors of anxiety and fear related to COVID-19.

Prior to analysis, data were screened for completeness. Missing data were minimal and handled using complete-case analysis.

### Ethical Considerations

The updated October 2024 version of the Declaration of Helsinki [46] was taken into consideration for this study, and explicit permission was obtained from participants through informed consent. This ensured the confidential use and processing of data in accordance with Organic Law 3/2018 on the Protection of Personal Data and Guarantee of Digital Rights [47] and Law 14/2007 on Biomedical Research [48]. All data will be duly safeguarded by the research team.

This study forms part of the IMPACTCOVID-19 project, which aims to evaluate the impact of the COVID-19 pandemic on the emotional well-being and psychological adjustment of the general population in Spain. The project was authorized by the Ethics Council and Research Committee of the Regional Government of Andalusia (reference PI 036/20-2)

Authorization to conduct the study was also granted by the Subdirectorate General for Institutional Relations and Territorial Coordination and the General Secretariat of Penitentiary Institutions of the Spanish Ministry of the Interior.

Participation was entirely voluntary. Before completing the questionnaire, all participants received verbal and written information regarding the objectives of the study and were informed that participation was anonymous and confidential and would have no effect on their legal, penitentiary, or health care status. Participants could decline participation or withdraw at any time without consequences. All participants confirmed their voluntary and confidential participation by selecting the corresponding checkbox on the informed consent form. The form additionally informed participants of the possibility of contacting the principal investigator if they required psychological support after completing the questionnaire; for this purpose, participants were invited to provide their full name and email address. Participants received no compensation for taking part.

## Results

### Descriptive Analysis of the Sample

The study sample consisted of 711 incarcerated adults aged 18 years or older from prisons in Spain (Table 1). Of these 711 individuals, 100 (14.1%) were women, and 611 (85.9%) were men. Participants ranged in age from 19 to 82 years, with a mean age of 40.41 (SD 11.67) years. Additionally, 19.3% (n=137) were married, 39.1% (n=278) were single, and 41.6% (n=296) reported another marital status (divorced, separated, widowed, or living with a partner). Furthermore, 67.5% (n=480) had children, while 32.5% (n=231) did not.

**Table 1.** Sociodemographic characteristics of the study sample and bivariate analysis of anxiety and fear related to COVID-19 among incarcerated adults in 3 Spanish prisons (Spain, 2022‐2023; N=711).

Variables	Total sample, n (%)	AMICO^a^ score (range 1-10), mean (SD)	Hypothesis testing, *P* value
Sex			<.01^b^
Female	100 (14.1)	6.57 (2.62)	
Male	611 (85.9)	5.42 (2.50)	
Marital status			.59^c^
Married	137 (19.3)	5.52 (2.72)	
Divorced	90 (12.7)	5.46 (2.63)	
Other	9 (1.3)	4.48 (2.23)	
Single	278 (39.1)	5.50 (2.53)	
Partnered	178 (25.0)	5.85 (2.46)	
Widowed	19 (2.7)	5.85 (2.23)	
Degree of sentence			.66^c^
First degree (closed regime)	12 (1.7)	6.11 (1.83)	
Second degree (ordinary regime)	685 (96.3)	5.58 (2.55)	
Third degree (open regime)	14 (2.0)	5.17 (3.28)	
Children			.03^b^
Yes	480 (67.5)	5.74 (2.56)	
No	231 (32.5)	5.06 (2.50)	
Receipt of a pension			.84^b^
Yes	115 (16.2)	5.61 (2.56)	
No	596 (83.8)	5.58 (2.55)	
Sharing a cell			.05^c^
Yes	508 (71.4)	5.32 (2.57)	
No	203 (28.6)	5.19 (2.50)	
Chronic illness			.002^b^
Yes	241 (33.9)	6.00 (2.48)	
No	470 (66.1)	5.37 (2.56)	
Substance use			.20^c^
No substance use	300 (42.2)	5.44 (2.62)	
Alcohol only	11 (1.5)	4.42 (2.42)	
Tobacco only	278 (39.1)	5.74 (2.47)	
Other substance or polysubstance use	122 (17.2)	5.68 (2.55)	

a AMICO: Anxiety and Fear of COVID-19 Assessment Scale.

b Mann-Whitney *U* test.

c Kruskal-Wallis test.

Concerning the degree of sentence classification, 1.7% (12/711) were in the first degree, characterized by more restrictive control and security measures (closed regime); 96.3% (685/711) were in the second degree (ordinary regime); and 2.0% (14/711) were in the third degree (open regime). A total of 16.2% (115/711) received some type of social or noncontributory pension, whereas 83.8% (596/711) did not. The data also indicate that 71.4% (508/711) of respondents shared a cell, while 28.6% (203/711) did not share a cell with anyone. Regarding substance use, 42.2% (300/711) reported no use of any substance, 39.1% (278/711) used tobacco, and 41.4% used other legal or illegal substances.

The mean score obtained on the AMICO was 5.58 (SD 2.55) points, with a range from 1 to 10. The bivariate analysis identified statistically significant differences only in relation to sex, with women presenting a higher mean score (mean 6.57, SD 2.62) than men (mean 5.42, SD 2.50; *P<*.01). For the remaining variables analyzed (marital status, degree of sentence, having children, receipt of a pension, and sharing a cell), no statistically significant differences were observed in the mean AMICO score, with similar values across categories (Table 1).

In relation to the findings derived from the use of the AMICO, Table 2 shows the mean scores and SDs for the 16 items that constitute the instrument. Overall, higher scores were observed for items related to concern about the infection of family members and friends (mean 8.11, SD 2.835), as well as the uncertain duration of the pandemic (mean 7.51, SD 3.130) and the possibility of death from COVID-19 (mean 7.14, SD 3.331).

**Table 2.** Descriptive statistics of the Anxiety and Fear of COVID-19 Assessment Scale–Inmates items assessing anxiety and fear related to COVID-19 among incarcerated adults in Spanish prisons (2022‐2023; N=711).

Items	Score (range 1-10), mean (SD)
I am very afraid of COVID-19.	5.48 (3.325)
I feel uneasiness when thinking about COVID-19.	5.76 (3.391)
I am very concerned about getting COVID-19.	6.29 (3.465)
The COVID-19 disease may cause death, and this worries me.	7.14 (3.331)
My hands sweat when I think about COVID-19.	4.07 (3.466)
I feel nervous or uneasy when watching news or stories about COVID-19 in social networks and other media.	5.53 (3.508)
I can’t sleep because I am worried about getting COVID-19.	3.61 (3.239)
My pulse races when I think about getting COVID-19.	3.94 (3.427)
Contradictory information about coronavirus in social networks and the media makes me feel anxious.	4.72 (3.514)
I have negative ideas when I hear or read any news related to the disease.	5.06 (3.470)
I am afraid any relative or friend may get COVID-19.	8.11 (2.835)
I am worried about how long the pandemic will last.	7.51 (3.130)
When someone coughs near me or I consider is too close to me, I am afraid of getting infected.	6.20 (3.429)
I am worried about being close to or assisting a person that has or may have COVID-19.	6.66 (3.464)
I feel sad or downcast when I think about the disease and the possibility of getting infected.	5.32 (3.559)
I feel anxious about going out, or considering going out, to fulfill my daily responsibilities (work, family, etc).	3.90 (3.400)

In addition, items linked to fear of contracting the disease and physical proximity to others showed elevated scores, as reflected in statements such as “When someone coughs near me or I consider is too close to me, I am afraid of getting infected” (mean 6.20, SD 3.429) and “I am worried about being close to or assisting a person that has or may have COVID-19” (mean 6.66, SD 3.464).

Conversely, the lowest scores were recorded for items associated with physiological anxiety responses, such as “I can’t sleep because I am worried about getting COVID-19” (mean 3.61, SD 3.239), “My pulse races when I think about getting COVID-19” (mean 3.94, SD 3.427), and “I feel anxious about going out, or considering going out, to fulfill my daily responsibilities (work, family, etc)” (mean 3.90, SD 3.400).

Taken together, these findings indicate that, within the prison sample, cognitive concern about contagion and the overall impact of the pandemic predominated over physiological symptoms of anxiety (Table 2).

### Regression Analysis

Regarding the association between sociodemographic and health-related variables and AMICO scores, the bivariate analyses revealed statistically significant differences according to sex, having children, sharing a cell, and the presence of chronic illness (Table 1). Women, inmates with children, those sharing a cell, and those reporting a chronic illness showed higher mean levels of anxiety and fear related to COVID-19. No statistically significant differences were observed for marital status, degree of sentence, receipt of a pension, or substance use.

A categorical regression analysis was performed to determine which sociodemographic variables were associated with anxiety and fear scores related to COVID-19 as measured using the AMICO. The resulting model yielded an *R*^2^ of 0.174, indicating that the variables included explained approximately 17.4% of the variance in the total score. Although the proportion of the variance explained was modest, the model was statistically significant (*F*_7,703_=6.845; *P<*.001), as shown in Table 3.

**Table 3.** Categorical regression model examining sociodemographic predictors of anxiety and fear related to COVID-19 (Anxiety and Fear of COVID-19 Assessment Scale total score) among incarcerated adults in Spanish prisons (2022‐2023; N=711).^a^

Variables	β coefficient	*F* test (*df*)	*P* value
Sex	0.214	16.939 (2, 703)	<.001
Having children	−0.049	1.308 (1, 703)	.25
Sharing a cell	0.112	6.918 (2, 703)	.001
Chronic illness	0.086	4.615 (2, 703)	.01

a Regression model fit: *R*2=0.174, Fisher *F*_7,703_=6.845, and *P*<.001.

With respect to the standardized coefficients, 3 variables showed a significant association with the total AMICO score: sex, sharing a cell, and chronic illness. Sex presented the highest β coefficient (β=0.214; *P<*.001), indicating higher levels of anxiety and fear among women than among men. Sharing a cell was also associated with higher AMICO scores (β=0.112; *P=*.001), as was the presence of a chronic illness (β=0.086; *P=*.01). Having children did not remain significant after adjustment in the multivariable model (β=−0.049; *P=*.25).

## Discussion

### Principal Findings

The present study contributes to the growing body of evidence on the psychological consequences of the COVID-19 pandemic by focusing on a population that remains largely underrepresented in the literature: incarcerated individuals. Overall, the findings suggest that the pandemic generated a notable emotional burden among inmates, highlighting the vulnerability of prison populations when confronted with large-scale public health emergencies. These results reinforce previous evidence indicating that restrictive environments, reduced autonomy, uncertainty, and concerns about personal and family well-being can intensify psychological distress during health crises [49].

The findings further suggest that emotional responses to COVID-19 within prisons are not distributed uniformly across individuals. Rather, they appear to be influenced by both personal and contextual factors, supporting ecological and psychosocial models that conceptualize anxiety as the result of interactions between individual characteristics and environmental conditions [20,50,51]. In correctional settings, these dynamics may be particularly relevant given the unique challenges associated with confinement, social isolation, and limited control over daily living conditions [19,52].

From a broader perspective, the study highlights the importance of considering prisons as priority settings for mental health surveillance and intervention during infectious disease outbreaks. The experience of the COVID-19 pandemic demonstrated that concerns related to health risks, uncertainty about the future, and the well-being of significant others may become especially salient among incarcerated populations. These concerns are consistent with theoretical frameworks emphasizing the role of perceived vulnerability, uncertainty, and mortality awareness in the development of anxiety responses during periods of crisis [51-56].

Taken together, the need for prison health policies that incorporate psychological support strategies alongside infection control measures is noticeable. Addressing the emotional consequences of public health emergencies within correctional institutions may contribute not only to the well-being of inmates but also to the overall effectiveness of institutional responses to future crises [12,57].

Our findings are consistent with those of international research documenting intensified emotional distress among individuals deprived of liberty during the pandemic, particularly as a consequence of suspended visits, health-related uncertainty, and perceived risk [58]. Studies conducted in different countries have reported similar patterns, including increased fear of contagion, concern for family members, and perception of institutional vulnerability, factors that have contributed to a widespread rise in symptoms of anxiety, stress, and depression within prison populations [51]. These elements have been identified as especially significant in settings with stringent restrictive measures, where prolonged isolation and reduced external contact act as triggers of emotional vulnerability [59].

The association between chronic illness and higher anxiety and fear scores is also consistent with previous evidence from both prison and community settings. Individuals living with chronic health conditions were repeatedly identified during the COVID-19 pandemic as being at increased risk of severe illness and adverse outcomes, which may have heightened perceptions of vulnerability and uncertainty. Within correctional environments, concerns regarding access to health care, continuity of treatment, and the potential consequences of infection may further amplify these emotional responses. Consequently, inmates with chronic illnesses may represent a particularly vulnerable subgroup requiring additional psychological and health support during public health emergencies [60].

Similarly, the higher levels of anxiety observed among women align with meta-analyses showing a greater prevalence of anxiety disorders in women under adverse circumstances [61]. Within the female prison population specifically, the literature identifies an increased risk of affective and psychological disorders related to factors such as greater exposure to past traumatic experiences, separation from children or other dependents, and the emotional burden associated with caregiving responsibilities [62]. These factors may have amplified emotional responses during the pandemic, thus helping explain the differences observed.

Elevated concern about infecting family members has also been documented in European and Latin American prison studies, where fear for loved ones’ health frequently exceeded fear for one’s own physical integrity [63,64]. This pattern is consistent with psychological theories suggesting that perceived threats to significant others can intensify fear due to the activation of attachment and social protection mechanisms [65].

Finally, the findings are in line with evidence from the general nonprison population, which also reported heightened anxiety in the face of uncertainty, risk of infection, and exposure to contradictory information [66,67]. However, in the prison context, these factors appear to be exacerbated by reduced autonomy, limited access to reliable health information, and a perceived lack of control over the environment, which may account for the high scores observed in several AMICO items.

### Contributions, Limitations, and Future Directions

This study makes several contributions to the perception of emotional distress in prison settings. The findings provide evidence on the factors associated with anxiety among incarcerated individuals and highlight the particular vulnerability of women, individuals with chronic illnesses, and those living in shared cells. From a practical and managerial perspective, the results highlight the importance of implementing targeted emotional support interventions, improving staff training in the early detection of anxiety symptoms, and promoting effective communication and organizational practices that support inmates’ psychological well-being during health emergencies.

Despite these contributions, several limitations should be acknowledged. First, the study relied on self-reported data, which may be affected by social desirability bias. Second, the cross-sectional design prevents the establishment of causal relationships among the variables examined. Third, a nonprobability convenience sampling approach was used due to the restricted access to correctional settings and the operational constraints inherent to prison research, which may introduce selection bias and limit representativeness. In addition, the absence of prior stratification may have resulted in imbalances across relevant subgroups, such as sex or other custodial characteristics, potentially affecting the precision of the estimates and the stability of the predictive model. Although female sex emerged as the strongest factor associated with anxiety and fear related to COVID-19, women represented a relatively small proportion of the sample. Therefore, the magnitude and generalizability of this association should be interpreted with caution and confirmed in future studies including larger and more balanced samples of female inmates. Accordingly, the findings should not be assumed to be fully representative of all incarcerated populations. Fourth, data were collected between May 2022 and February 2023, a period marked by the progressive easing of COVID-19 restrictions and the transition toward a postpandemic context. Therefore, the level of fear observed in this study may not fully reflect current perceptions of COVID-19–related fear among incarcerated populations. Finally, relevant psychological variables such as perceived stress, coping strategies, and social support were not included. Although the CATREG model explained a meaningful proportion of the variance in anxiety and fear related to COVID-19, the inclusion of these variables could have further improved its explanatory capacity. In addition, the number of inmates who declined participation was not systematically recorded, preventing the calculation of a response rate and limiting the assessment of potential selection bias.

### Conclusions

The findings of this study show that more than half of the evaluated Spanish prison population presented high levels of anxiety and fear related to COVID-19. This prevalence confirms that the pandemic had a substantial emotional impact on a population characterized by structural vulnerability, restrictions on personal autonomy, and limited access to information and social support.

The bivariate analysis and the categorical regression model identified clear sex differences, with women exhibiting significantly higher scores, as well as notable effects associated with sharing a cell and the presence of chronic illness. These results underline the need to consider differential factors, both structural and personal, when assessing emotional distress in prison environments.

The data obtained provide an up-to-date and valuable overview for prison institutions and public health authorities, offering empirical evidence on the emotional needs of incarcerated individuals during health crises. Consequently, the findings support the importance of developing specific preventive and therapeutic interventions, with particular attention to women, individuals with chronic illnesses, and those living in shared cells.

Finally, the results highlight the relevance of promoting further research that integrates psychological, social, and environmental variables, as well as longitudinal studies that allow for a deeper understanding of the evolution of emotional distress among people deprived of liberty following public health emergencies.

## Supplementary material

10.2196/88699 Checklist 1 STROBE checklist.

## Abbreviations

AMICO: Anxiety and Fear of COVID-19 Assessment Scale
STROBE: Strengthening the Reporting of Observational Studies in Epidemiology
